# 2122 Perspectives on increasing competency in using digital practices and approaches to enhance clinical translational research: A qualitative study

**DOI:** 10.1017/cts.2018.220

**Published:** 2018-11-21

**Authors:** Katja Reuter, Kelsey Simpson, Namquyen Le, Ricky N. Bluthenthal, Cecilia M. Patino-Sutton

## Abstract

OBJECTIVES/SPECIFIC AIMS: The use of digital practices and approaches can potentially increase the quality and efficiency of all phases of the traditional clinical translational research (CTR) process. The purpose of this qualitative study was to describe key stakeholders’ perspectives on the need to: (A) formalize training in digital practices and approaches among CTR trainees; and (B) develop an aligned educational framework that defines core competencies, educational methods, and evaluation metrics. METHODS/STUDY POPULATION: Participants (n=66) were recruited via email from June to November 2017 using purposive and snowball sampling methods across 4 groups: (1) English speaking national and international experts from academic and private sector institutions with working experience in using digital practices and approaches in research (n=36), (2) CTR educators (n=8), (3) CTR trainees (n=13), and (4) Members of the Southern California Clinical and Translational Science Institute at the University of Southern California (n=9). Online focus groups were conducted using a semi-structured, open-ended interview guide through Google Hangouts and a conference call interface. Sessions were recorded and transcribed verbatim, and 2 research team members performed independent content analyses to identify before and emergent themes using an inductive analytic approach. Kappa was calculated for inter-rater agreement and repeated until agreement was at least 0.70. RESULTS/ANTICIPATED RESULTS: Participants’ average age (41.2 yrs, SD 9.26), gender (59% females), non-Hispanic (97%), race (72% White), and doctoral degree (67%). In total, 85% reported experience in teaching digital practices and approaches in research, although 70% were currently not teaching in this field. Participants reported that complementary teaching in digital practices and approaches across the 15 Clinical and Translational Science Award (CTSA) CTR competency areas was relevant, especially in literature review, research implementation, statistical approaches, biomedical informatics, regulatory support, responsible conduct of research, scientific communication, translational teamwork, cross-disciplinary training, leadership, and community engagement; and less so in literature critique, study design, sources of error, and cultural diversity. Additional competencies were identified, for example, online study recruitment, crowdfunding, team and project management, scholarly impact metrics (Altmetrics), ethical and regulatory guidance for conducting research using digital approaches. Five main educational practices were identified including online training sessions, flexible on-demand modules, in-person consultations and training, and project-oriented hands-on workshops. Among the identified challenges were the need for clear metrics in order to evaluate such a training program. DISCUSSION/SIGNIFICANCE OF IMPACT: There was consistent support for a structured program to help CTR trainees to develop competency in digital research practices and approaches. Our results indicate that an education program focused on digital practices and approaches should include a step-wise approach to meet different research and training goals, allowing attendees to increase their awareness and specialized hands-on practical experience.

